# Book Reviews

**Published:** 1821-05

**Authors:** 


### CRITICAL ANALYSES

OF

### RECENT PUBLICATIONS, IN THE DIFFERENT BRANCHES OF MEDICINE AND SURGERY,

### In the Literature of Foreign Nations.

---

Πατρίδες ἄρα
Ανδράσιν, ὦ πατραῖς ἄνδρες, ἀγαλλόμεθα.

---

*Du Siége et de la Nature des Maladies; ou, nouvelles Considérations touchant la veritable Action du Systême Absorbant dans les Phenoménes de l'Economie Animale.* Par M. ALARD, D.M.P.; Chevalier de la Legion d'Honneur; Médecin en chief-adjoint de la Maison Royale de Saint-Denis; Médecin Consultant des Succursales de cette Maison; Médecin Honoraire des Dispensaires; et Membre de plusieurs Sociétés de Médecine nationales et étrangères. 2 tomes, 8vo.; de 367 et 577 pp. Baillière, Paris, 1821.

Morborum omnium unus et idem modus est: locus vèro ipse eorum differentiam facit.

HIPPOCRATES, *de Flatibus*, § iv.

THIS work owes its origin to the views which broke upon the mind of the author, after he had, in the year 1800, been engaged in translating into French Dr. HENDY's "Observations on the Glandular Diseases of Barbadoes." He was astonished, he says, when he saw, in the description of the English author, the history of a disease whose seat was evidently in the absorbents, whilst its symptoms and progress were analogous with those of several affections which had been attributed to various organs and another order of vessels. At first, his notions of this disease were only those at that time generally prevalent; one of which was, that the peculiarities in its phenomena were the proper effects of the particular climate and soil of the island in which it was endemical; but, on regarding it more attentively, he inferred that an affection which prevailed especially in seasons productive of inflammatory diseases,—which gradually became endemic as that atmospheric constitution became established,—and which alternated sometimes with fevers, and at others with inflammation of the viscera,—required for its production only a concourse of circumstances which every climate might present. This notion led to a series of researches, the results of which were published, in 1806, in a work of which the title was "A History of a Disease peculiar to the Lymphatic System," but which was afterwards changed to that of "A History of the Elephantiasis of the Arabs;" and at the same time that the author's inquiries enabled him to recognize the existence of the same malady in Asia, Africa, America, and even in Europe, he was again struck by the great number of relations and points of analogy which it presented with several other affections; and these relations and analogies induced him to consider the absorbent system as a structure having an importance in the animal economy very different from that which had hitherto been imagined. From this time the horizon of his views extended before his eyes, and he soon perceived that no precise knowledge of the extent of the disorders dependant on this system was yet possessed; and that he was in the way for attaining some discoveries that would reflect a new light on the proximate causes of diseases in general. Since that time, he has done what his clinical experience, literary researches, and powers of reflection, would enable him to effect towards the attainment of the object here alluded to; and the work we are about to take into consideration presents the general results of his labours.

The author, in his Preface, endeavours to obviate some prejudices which he supposes to exist against such a work, and to conciliate the minds of his readers to its principal object. "A book of theory," he says, "a book on the proximate causes of diseases, is brought forth very incongruously in an age when the disposition given to the minds of medical students leads them only to empiricism. I venture to believe, however, that a little reflection will lead to notions more conformable with the true interests of science. The illustrious Professor of the School of Paris who, one of the first, turned the minds of his contemporaries from theoretical speculations, reprobated only vain and futile theories, composed, almost solely, of vague and insignificant terms which passed current in the ancient schools. It appears to me that he has been but ill understood, when it has been supposed that he intended that we should constantly observe diseases without ever occupying ourselves with considerations respecting their origin." It is, he adds, "undoubtedly necessary to observe, and to observe well, facts; but these facts would remain barren, if we did not trace their relations, and thence form some general inferences respecting them, without which there can be no science."

Much of the declamation against theory with which they are pestered in France, as well as we are in England, has, it appears, then, arisen from a vague employment of terms: some men of good judgment have used the word *theory* when reprobating, not a general expression of inferences immediately drawn from facts, but conclusions founded, more or less extensively, on inferences raised on supposititious bases;* and then certain persons who are very apt at echoing, —and who are qualified to do nothing else than echo,—the expressions of men of place or merit, apply it to all sorts of reasoning; whilst their own conduct, to complete the absurdity of the matter, is such as is described so exactly and laconically in the cited passage introduced at the commencement of the review of the work of Mr. Bégin.

The work of Dr. Alard is constituted of two distinct parts: the one treating of the distribution of the absorbents, and the part those vessels perform in the vital functions in the state of health; the other, of their action in the phenomena of diseases. The anatomical and physiological disquisition occupies the whole of the first of the two volumes

* The word *hypo*-thesis expresses very well (in conformity with a general admission in modern languages,) this sort of reasoning; and it is a pity that all writers will not employ it where it is necessary.

into which the work is divided; and it has been rendered thus extensive, from the author, as it appears, having thought it prudent to display the bases of his inferences, and discuss the obvious objections to his conclusions, in such a manner as to relieve any of his readers from the necessity of seeking elsewhere for the most important of the facts which appear to support, or to oppose, the system of pathology which he has here developed. In the analysis we are about to give of this work, we shall suppose the reader to be informed of many things of which the author has considered it proper to produce detailed observations, but which are familiar to all physiologists; as well as of several inductions from facts, equally well understood, that he has not advanced without a narration of the evidence on which they are established.

Dr. Alard commences his work with the following proposition:—"*That which constitutes the basis of the body of man and of the most part of animals, is a tissue of vessels of diverse nature. The hardest parts themselves are but a vascular assemblage: their hardness depends only on the matter contained in the vessels, as the fleshy appearance has no other cause than the presence of blood, or of a sanguineous fluid, in the interior of those canals.*"

In support of this proposition, the author first notices the observations of Haller, King, Ruysch, and several other and later anatomists, which tend to show that the smallest perceptible portion of the structures of the animal body, even though, using the expression of Haller, it be no bigger than a grain of sand, is found, on examination by the microscope, to present a multitude of small vessels, different orders of which are filled with their proper fluids. The failure of injections to demonstrate this mode of structure in the bones, the brain, and some other parts, in first trials, was considered to present an objection to the generality of the inference above stated: but, as early as 1739, a French academician, on examining, with the aid of a good microscope, bones tinged with madder, found them to present, first a tissue of white fibres, then one of a reddish colour, under this a third, and then a fourth, of deeper-coloured fibres; so that the bone was, throughout the coloured part, wholly penetrated as it were by a natural injection. Some preparations of the bones, made by Scarpa, and preserved in the museum at the University of Pavia, seem also to show that the basis of the osseous structure is nothing but an aggregate of vessels. The brain was found by Leuwenhoek to be constituted, as far as the powers of the microscope could inform his sense of vision, wholly of a congeries of vessels filled with various fluids. These observations of Leuwenhoek were soon afterwards supported by others of a similar kind, made by Vieussens. We noticed the principal arguments for the application of the same general inference to the structure of the eye and several other organs, as well as the membranes formed in inflammations, when we gave an account of the Dissertation of Dr. Felici on this subject, in a late Number of this Journal. The author's researches on this point have been very extensive: he has, indeed, made himself acquainted with every thing of importance that has been advanced in support of the proposition with which the chapter commences. It does not, however, come within our views to notice the observations in detail; as they are amongst those which may be supposed to be generally understood. Some facts may, however, be adduced respecting the tubular structure of the muscular fibres, —which was first asserted by King, (in a paper published in the Philosophical Transactions,)—that will, perhaps, be novel to some of our readers.

After Blancardi had, as he asserted, injected the muscular fibres by means of the arteries distributed to them, Muys, on throwing warm water into the crural artery of a lamb, perceived the muscular fibres lose their colour and become entirely white: on passing, then, a coloured fluid into the artery, he found the muscles assume the colour of the liquid, and, on examining them attentively with the microscope, he was satisfied that the smallest fibres of the muscles were filled with the liquid; whilst no vestige of it appeared in their interstices. This is conformable with what is stated by Haller, in his commentary on the Prelections of Boerhaave; when he says, that "water injected into the coronary artery returned by the vein, at first coloured with blood; but it was gradually seen to become paler and paler, until it was quite colourless. The heart, preserving its form, had become as white as the stomach or the bladder." We shall conclude our observations on this subject, with remarking that, notwithstanding the evidence presented by the facts above detailed or alluded to, many eminent anatomists of the present day believe in the existence of a simple laminated texture, forming the base of the cellular and other membranes; and that, whilst such a texture is intimately interwoven with vessels, which, especially when distended by injections, may appear to occupy it wholly, there yet remains a membranous expansion, not of a tubular nature. With respect to the tubular structure of the muscular fibres, we have, also, to remark, that Mr. Carlisle has stated (in his Croonian Lecture for 1805,) that he can distinctly see an ultimate muscular fibre, which appears "as a solid cylinder, the covering of which is reticular membrane, and the contained part a pulpy substance irregularly granulated." We know not, however, what to believe on this subject,—the statements of men of eminent talents are so incongruous: for Leuwenhoek and Hook compare the ultimate muscular fibre to a string of pearls; Prochaska says it is as fine as a gossamer film, and extends throughout the whole length of a muscle; whilst Haller, admitting its filamentous character, says it extends only to a short distance along a muscle; Muys asserts that it has a knotted appearance; Blancardi describes it as being contracted here and there; whilst Lecat says that it looks somewhat like a knotted reed: the greater number of anatomists, and amongst them Cuvier, agree, however, in stating, that the utmost powers of the microscope present it to us in the form of a solid cylinder, of a filamentous character, similar to the fibres which are cognizable by the unassisted visual powers.

With respect to the vascular nature of the ultimate muscular fibres, we may add the remark, that some physiologists have regarded the fact that the muscles, in cases of death from asphyxia, have been found of a brownish-red colour, whilst the blood in the arteries was of a deep-purple hue, as a proof of the combination of colouring matter with the muscular fibre itself; as, did the colour of the muscles depend on blood circulating through them, they must assume the purple hue of the blood. But this inference is not irrefragable, even were it not opposed by the fact that muscles may be washed white,* by injecting water into their arteries. The ultimate fibres of a muscle may derive their colour from blood circulating through them, and yet preserve their ordinary hue when the larger vessels are filled with purple blood, in virtue of that law which causes certain vessels to admit only certain kinds of fluids to enter them under natural circumstances. This law was well appreciated by Bichat, in his reasonings on the functions of his capillary system, when he endeavoured to explain why those vessels refused to admit the red blood unless their vitality had undergone some modification; and it is somewhat extraordinary that he did not apply it to the condition of the muscular fibres in the case of asphyxia.

The second chapter commences with the proposition that "*the vessels which compose the basis of the bodies of men and animals communicate throughout with each other.*" Malpighi, the author remarks, appears to have first perceived the communication which exists between the arteries and the veins, by the aid of the microscope. His observations were afterwards supported by those of Leuwenhoek, who observed, besides, that it is impossible to determine where the arteries end and the veins begin. This intimate connexion throughout the body, appeared to him to be so well proved that he could conceive no other way of accounting for the secretions than the admission of a transudation of the fluids through the parietes of the small arteries. The observations of Leuwenhoek were made on fishes or amphibious and cold-blooded animals; and, therefore, some doubts were entertained respecting the mechanism of this part of the economy in man, or in hot-blooded animals. The fact of such a mode of communication of the arteries and veins in animals of the latter kind, was, however, soon established by the observations of Cowper; and subsequently by those of Cheselden, Haller, Spallanzani, and numerous later inquirers. The passage of injections from the arteries into the veins, in almost every part of the body, was shown by Ruysch, De Graaf, Vieussens, Haller, Mascagni, and has become a familiar experiment in every dissecting-room.

The communication of the larger blood-vessels with the lymphatics, has not been proved by an equal extent of evidence. The fact of such a communication has, however, been well established by Cruikshanks and several other anatomists.

But, besides the vascular lymphatics capable of attaining a size which renders them perceptible to our eyes and permits us to trace them with our instruments, there is another series of vessels, extremely small in diameter, which anatomists have been accustomed to call by

* Other physiologists than those above named have stated that this may be effected; but Gorter seems to stand alone, when he asserts that a muscle thus washed white—pale as a membrane, he says,—will preserve the faculty of contracting on being excited.

different names, in conformity with their supposed uses; and which were regarded as arterious by Ruysch, because he injected them by the arteries; as lymphatics, by Cruikshanks and Mascagni, because they injected them by the larger lymphatic vessels; and as venous by Dr. Ribes, because he has injected them by the veins. These circumstances, and the results of experiments on the absorption of fluids placed in contact with various surfaces, seem to warrant the inference that the sanguineous vessels are connected, by their parietes, with vessels which are distributed about the body in the utmost abundance, and developed on all the surfaces, exterior and interior; and which, though they generally carry only colourless fluids, transmit red blood under certain conditions.

The influence of the vital powers in preserving the vessels last alluded to from the passage of red blood, is well shown by some experiments made by Professor Buniva.* He had obtained some curious results on injecting the arteries of dead animals with blood diluted with water; and, wishing to try the effect of a similar injection in a living animal, he tied the axillary artery of a calf, opened the vessel below the ligature, and urged the injection into it with considerable force. The liquid entered with difficulty, and presented no sign of its presence on the exterior of the limb. The spinal marrow was then divided near to the head: the heart and arteries almost instantly ceased to pulsate; the animal ceased to live; and, as suddenly, the diluted blood which remained in the syringe passed with rapidity into the limb, and showed itself on the surface, as it had done in his former experiments with injection of the same fluid. Some of the experiments here alluded to were made on the human body, where the injection, too, was thrown into the aorta; and, as these presented the greatest variety of interesting phenomena, we shall transcribe an account of their results on subjects of this kind.

The skin generally assumed a blood-red colour, whilst the palms of the hands had a fine rose tint; a blistered surface became tumid, reddened, and covered with drops of blood; in certain parts,—the cheeks, for instance,—a bloody sweat broke out; the liver and the spleen swelled, and became so reddened as to present the appearance of violent inflammation; the stomach was so red as to have the semblance of a muscle; the intestines showed a similar aspect, whilst bloody fluid oozed from their parietes; the conjunctiva was as red as it is in ophthalmia; the iris, the retina, the sclerotica, the choroides, and the capsules of the aqueous and vituous humours, partook of the same colour; as did also the bones and periosteum. The phenomena just described, and especially the diversities in the results of the experiments when living and dead animals were the subjects of them, prove, in the most decisive manner, that during life the red part of the blood is retained in its proper vessels by the laws of vitality, rather than by a want of capacity in the lymphatics to admit of its reception into these vessels; whilst they demonstrate the continuity of the vascular system in all its parts.

* Published in a memoir read to the Institute in 1799.

The author next discusses the question of the existence of any organ or order of vessels continuous with the extremities of the arteries and the origin of the veins, and in which the circulation is independent of the heart. Although he considers that this question had already been satisfactorily replied to, in the negative, by the facts already cited from Malpighi, Leuwenhoek, Cowper, Vieussens, and others, of the continuity of the arteries and veins, he thinks it proper to notice the results of several experiments which serve to establish the propriety of this decision. He remarks, that Leuwenhoek saw signs of the impulse of the heart communicated even to the largest veins; that Hales observed the motion of the blood accelerated, by each systole of the heart, in both the arteries and the veins of the lungs of frogs; that Spallanzani has proved, by experiments, that the rapidity of the movement of the venous fluid is increased during the systole of the heart, and lessened in the diastole, in several species of lizards, and that, not only in a few small veins, but in an innumerable quantity, whether the circulation was effected with the greatest rapidity or in a languid manner; and he concludes with relating the following experiment of Magendie. This physiologist laid bare the crural artery and vein of a dog, and put a ligature round the whole of the limb excepting these two vessels; he then tied the crural vein, and opened it below the ligature: the blood sprang from it in a continuous jet, of considerable volume. He then compressed the artery: the jet from the vein continued from a few instants, but it diminished as the artery became devoid of blood, and ceased entirely when it was completely empty: although the vein was "gorged" throughout its whole length, blood no longer escaped from the opening in it. The compression of the artery was removed at this instant; the blood entered the vessel, and the jet of blood from the vein was immediately renewed. Similar results occurred in the several repetitions of this experiment that were instituted.

Dr. Magendie, wishing to show the supposed fact in question in another manner, introduced the extremity of a syringe filled with warm water into the crural artery, and gently propelled the piston: the blood in the vein instantly escaped, at first alone, and then mingled with water, in a jet which was more or less considerable according as more or less force was exerted on the piston of the syringe.

To this evidence of the existence of a continuous volume of blood from the arteries to the veins, Dr. Alard joins some physiological considerations, tending to show, as he infers, the danger that must result to the economy were the motion of the blood influenced, to any considerable degree, by the capillary vessels independently of the heart. "The movements of this '*circulatory circle*,' (he says,) must be very regular, in order that health, and even life, may be sustained; for it is absolutely necessary that the blood which, in the time of one pulsation, has passed from the arteries to the veins, should repass in the same quantity, and in an equal period, from the veins to the arteries. If the transmission were not to take place in this due proportion, it is obvious that engorgement of the veins would ensue; and, on the contrary, the same condition of the arteries would happen if, in the time of a second pulsation, more blood passed from the veins to the arteries than had passed from the arteries to the veins. Were this relation of the course of the blood to exist for any considerable length of time,—though the blood may accumulate in the veins to a certain point without much alteration of the health,—the circulation would certainly be disturbed: and how could this danger be absent for an instant in a system of which the parts were unconnected, or of which the two portions, although united by a common centre, should yet be regulated by different laws, and which should propel their fluids by two impulsions which could not agree? Such, indeed, would be the sanguineous circulation, if there really existed a system of vessels intermediate to the arteries and veins, which was free from the influence of the action of the heart, and in which the blood would enter in a multitude of diverse ratios, according to the casual modifications of sensibility." This conclusion of Dr. Alard, it cannot be concealed, is founded on an assumption that is merely gratuitous, and which may not appear to be so plausible to others as it is to himself. Many physiologists will think that it does not appear so necessary a result that the circulation must be commonly disturbed, were it influenced by an order of vessels intermediate to the larger arteries and the veins, and independent of the action of the heart. The preservation of a due relation between the two motive powers, would not be incongruous with several other relations in the economy, as that of the iris with the retina, that of the respiratory actions with those of the heart, by which the former are executed quicker or slower, in conformity with similar changes of the motions of the heart.

Dr. Alard adduces several other considerations in support of his opinion, though he notices but few important facts that had not been brought forward, with similar views, by Dr. Charles Parry. Dr. Alard admits, however, with the generality of physiologists who regard the heart as the predominant agent of the circulation throughout its whole extent, that the arteries and the veins possess "a moderate degree of sensibility; such as is qualified, at the utmost, to second the impulsion given by the heart." The question here noticed is one that is not likely to be soon, or even ever, settled. The cases in which satisfactory evidence of a distinct propulsive motion of the capillary vessels is manifested in warm-blooded animals, are instances of deviation from the ordinary state of health; and, considering the diversities of judgment of different men, even those of the best disciplined minds, on all inferences from merely analogical reasoning, it can hardly be expected that diversities of opinion will not be entertained respecting the propriety of regarding the manifestation of effects only in states of disorder as evidence of their existence in that of health. Perhaps the case the least remote from health in which motion of a small artery, distinct from that of the heart, has been observed, is that related in the Dissertation which gained the Jacksonian prize in the year 1813. The author of this Dissertation says, that the dorsalis pollicis in his right hand is large and conspicuous, so that the action of it may be seen as well as felt. On producing an inflammation of the skin covering this artery, by friction, the number of its pulsations, in a given time, has been greater than those of the radial artery of the same side: thus, he says, "the pulsations of the arteries generally were at seventy; the dorsalis pollicis has been irritated, it has beat eighty-four times in a minute; the radial artery was immediately examined, and was found to beat seventy-two strokes in a minute; the examination was again transferred to the dorsalis pollicis, the pulsations of which still continued at the rate of eighty-four in a minute, the vessel being visibly enlarged by the friction."

We advert to the fourth chapter of the work before us, which is thus entitled: "The humours are elaborated, without the sanguineous *circulatory circle*, by the absorbent vessels. Properties of these vessels." After remarking, in conformity with the inferences drawn in the preceding chapter, that the circulation of the blood should be regarded as "a physical rather than a medical phenomenon," the author observes, that "this circulation of the blood in the arteries and veins would be entirely useless for the maintenance of life, if there did not pass from those vessels "*rivulets*" which irrigate and nourish the invisible tissues of the parts adjacent. These rivulets are not formed by simple jets of extravasated fluids: pellucid canals, charged with the irrigation of these parts, every where spring from the arterial parietes. The existence of these canals is doubted at the present day, after having, for a long time, been admitted as a demonstrated truth. An opposite of this sort in the opinions of men depends on the extreme tenuity which these tubes always preserve, and especially to a degree of pellucidness which renders them invisible when they are empty or contain only fluids diaphanous as themselves, and which, also, permits them to assume the red colour whenever they are penetrated by blood; thus causing them to be often confounded with proper sanguiferous vessels. This double source of illusion renders the study of them one of the most difficult points of anatomy, and must throw in the way of those who devote themselves to it inevitable subjects for contradiction. We may, however, assemble, a series of facts dispersed in a great number of works,—facts too much neglected by the moderns, and which, when united, form an imposing collection of proofs in favour of the existence of these supposed tubes, parting from the parietes of the blood-vessels; which it would be difficult to avoid admitting, even though we could not materially demonstrate them, if we would explain, in a plausible manner, either the phenomena manifested by the nutritive functions, or the symptoms of the greater part of diseases." The propriety of these inferences will not be denied, we think, by any physiologist; the only point for disputation being, whether these pellucid vessels,—lymphatics, or exhalants, as they are most commonly termed,—spring from the sides of the arteries, or are expansions of the extremities of some of them which are not continuous with veins: for, whilst the fact of this communication of the arteries and veins has been admitted, as applied to the vessels, to a certain extent, it has been conjectured that there are other arteries which finally terminate in an expansion of exhalant vessels, admitting only colourless fluids, whilst the red parts of the blood are transmitted by the nearest proper arterial ramifications.

This last question is the subject of discussion in the fifth chapter of the work before us, which commences with the proposition that "the capillary system, such as it was conceived to be by Bichat, has no real existence." We shall pass over the evidence in the last chapter, tending to establish the inference of the existence of the pellucid vessels above alluded to: our limits will not permit us to give a comprehensive account of it; and it is, besides, detailed in works familiar to those versed in the study of anatomy. We could not do justice to the reasonings of the author on this evidence in an abstract, but we may remark that he has supported his inference in a very forcible manner. His arguments against the views of Bichat on the point just alluded to, serve to show that the functions attributed by this physiologist to his imaginary particular system of vessels, and the apparent laws by which those functions are regulated, are conformable with those of the absorbent system generally, and incongruous with those of the proper sanguiferous system.

This discussion is continued through the subsequent chapter; in which he endeavours, more especially, to prove that "the exhalant and cellular systems are only dependences of the absorbent system."

The subject is discussed, in a more general manner, in the seventh and eighth chapters; the objects of which are to show, that "the active part of the parenchymatous substance of the organs is composed only of absorbent vessels;" and that "the absorbents are the sole agents of nutrition and growth, as well as of decrease and decripitude." We shall endeavour to give a view of the author's opinions on these subjects in a concise general abstract, without following him through his discussions; for, by proceeding, as he has done, from proposition to proposition, discussing each singly, he has been obliged to enter into many repetitions, which it is not requisite that we should detail. One series of repetitions in which he indulges, and which we may just notice here, is that in which he endeavours to show that the nerves, the muscles, the cellular texture, and, in a word, all the soft parts of the body, are constituted of a congeries of minute vessels, variously arranged, containing fluids of diverse kinds, in virtue of the different vital properties, or modes of sensibility, which those vessels possess in the several organs or peculiar structures. These vessels, Dr. Alard believes, possess every where the power of contraction and dilatation, and receive diversities of fluids as they are differently excited by external agents; as we see, very well, in those of the conjunctiva of the eye, which, when stimulated, admit, or, using the author's expression, *pump* the red blood from the arteries with which they communicate. The only difference between the author's view and that of Bichat on this point, seems to be that the former considers the functions of these vessels to be similar to those of the larger series which have ordinarily been termed absorbents,—a notion which has led him to regard the whole as one system, to be designated by one term; whilst Bichat considered them to be expressly analogous to the arteries, and indeed as the final expansions of these vessels. Bichat, however, it must be remarked, whilst he contemplated the abundant distribution of capillary vessels in every structure, did not suppose but that there existed also a sort of simple membranous expansion or laminated texture, which formed the bases of the parenchymatous structures in which those vessels are so copiously distributed, as well as of various other modes of simple fibrous arrangement.

The view of Dr. Alard,—that of minute pellucid vessels, springing from the parietes of the small arteries; distributed in every point of the body; conveying different fluids and performing different organic functions, according as their vital properties are modified; having corresponding vessels, which may be said to spring from the most intimate texture of the organs, uniting into larger tubes, forming in some instances long continuous canals, (generally denominated absorbents,) in others running to be inserted in the parietes of veins, —is one that is calculated to explain, more plausibly than any other, the mechanism of the distribution of the fluids for the purposes of the organic functions, and several of the most remarkable phenomena manifested in disordered states of those functions; whilst it does not want plausible arguments in its support, (as Dr. Alard has well proved,) and is, besides, calculated to obviate the difficulties which have been presented by the results of the experiments of Hunter, Magendie, Brodie, and other eminent physiologists, on the mechanism of absorption. Still, it must be acknowledged, the author's inferences are founded, principally, on merely plausible suppositions; but these suppositions have lately received a very important degree of support in the discovery of Dr. Fohmann, of Heidelberg, of a communication of the lymphatics of the intestines with the mesenteric veins. It requires not a great stretch of analogy to infer—considering the evidence in favour of the existence of the pellucid vessels in question, presented by the observations and experiments of Ruysch, Cruickshanks, Ribes, (see page 415,) and several other anatomists,—that a communication which exists between a series of those vessels so large as to be palpable to the senses, exists also in such as are too minute to be thus recognized.

It may be here asked: if the basis of all the organs is but a congeries of vessels, allied to absorbents by their nature, how is it that certain organs execute functions so different from those of other parts; and, as an example, how is it that absorbents, arranged as a muscle, possess the power of contracting with such force as is witnessed in this species of organ? To this the author would reply, that the same order of vessels possesses peculiar properties superadded to the general characteristics of those vessels, in consequence of diversities in their alliances, arrangements, and the fluids they contain; and, in respect to the contractility of the muscles, he observes, that this faculty is the proper quality of absorbents; so that, instead of saying that absorbents possess muscular properties, it would be better to say that muscles possess, in an eminent degree, the inherent property of those vessels,—that of contracting whenever they are submitted to exciting agents; and if, using his own expressions, " muscles are provided with the contractile power in so high a degree, it is not to the peculiar nature of their fibres that they owe this prerogative, but to the manner in which those fibres are disposed in the intimate structure of those organs; to the union of those innumerable bundles of parallel fibres, the contraction and agency of which all the surrounding parts concur in favouring."

By admitting this hypothesis, we readily dispose of the difficulties which beset the questions of the structure of the urethra, uterus, iris, and choroid processes; and it is, in the structure of some polypi, &c. of course, applied to such a purpose by the author. The uterus, in his views, becomes muscular during pregnancy, because its vessels assume then that arrangement which constitutes a muscle; and it ceases to be muscular after parturition, because it then loses this particular arrangement. It is unfortunate, however, for this view of the subject, that no such arrangement as that on which the author's inference is founded is recognizable by our senses, in any stage of utero-gestation. The absorbent vessel, then, is, according to Dr. Alard, the seat of irritability, whilst it is the most simple, and only essential, part of animal organization. The seat of irritability is a point that has so perplexed the most eminent physiologists, that one could almost wish that he could persuade himself,—as, in the want of sensitive evidence in this case, we must incline our belief, more or less, to some hypothesis or other,—to admit the notions of Dr. Alard. Finding that organization devoid of nerves, (as well as our senses can inform us,)—as in the case with some polypi and the vast class of microscopic animals,—possess even more delicate excitability than the organs of man, we must agree, with Haller, that irritability is not the exclusive property of nerves; but then we are, by the same reasonings, inclined to reject his opinion that it is the exclusive property of muscular fibres, when we find that animals are, as Albinus remarked, more irritable in proportion as they more nearly resemble a gelatinous mass devoid of any distinction of parts; and thus we are induced to believe, with the physiologist just named, that this jelly is the proper seat of irritability: and here Dr. Alard will come forward, and say that this moving jelly could not possess the power of contraction and locomotion if it were not organized, if it were not enclosed by a diaphanous cellular tissue which has selected it from various alimentary matters, which causes it to flow and reflow in the interior of this tissue, and at last rejects portions of it in order to replace them by others. This cellular tissue, then,—composed of filaments more or less parallel to each other, and differing only by its tenuity from that of larger animals,—is evidently the means of sensible contractions, since it is the only solid part existing in the most simple mode of animal organization: and we may even see, as we rise in the scale of animal beings, some of these filaments merely placed in juxta-position, without being united with the least cellular texture, from the first type of the muscular organs, as is manifest in certain insects. Even the human embryo is similar in its apparent structure, in the early stages of its existence, to the more simple forms of animal organization; being constituted only of a gelatinous mass, interspersed with absorbent vessels, the orifices of which open externally, instead of into an internal cavity as in the medusa.

The view which Dr. Alard has taken of this subject, is conformable with the simplicity which some men take delight in imagining to exist in the means employed by nature. In inorganic bodies, attraction is sufficient in order that each portion of matter may find its proper place in the vast universe. In animals' bodies, sensibility produces the innumerable phenomena of vitality. The means on which absorption depends become, in muscles, in consequence of a particular disposition of parts, the powerful motive agent by which an animal transports itself from one place to another. The same vessels which are every where else moveable, are in the bones gorged with calcareous phosphate, in virtue of the same principle, and thus become points of support and levers to the motive organs previously designated.

As the absorbent system is the part first formed in the fetus, so does it predominate in the body during the earlier periods of life, or until the growth is completed: it then gradually loses its vitality; the fluids formerly absorbed by the exhalant order of vessels, are either retained in the arteries, or they are returned into the torrent of the circulation by the veins; the absorbents decay in their structure as well as in their functions; the glands cease to appear reddish and pulpy; the fluid, with which they had ordinarily been filled, disappears, and the organs themselves are often obliterated with age, and a plethora of the blood-vessels is the result. The bones also present signs of this species of change in the economy. The exhalant absorbents had carried to these structures the gelatinous and calcareous matter on which their cohesion and formation depended; the resorbing order of vessels gradually remove those vessels in old age; as this period of life extends, the bones become less hard and less weighty than in adult age; the compact substance of them diminishes in thickness; the cellules of the spongy tissue become more ample, the medullary cavity enlarges; the calibre of their blood-vessels increases; and, in a word, their calcareous matter returns into the torrent of the circulation, and is deposited in various parts; thus producing ossifications which often hasten the arrival of the final period of human existence.

The successive changes in the human body above slightly alluded to, are discussed in a very particular manner by Dr. Alard, who finds in them many phenomena, which he employs, with much force, in support of his views of the functions of the absorbent system in the animal economy. We can only recommend the more studious part of our readers to peruse his work: our limits oblige us to pass over the important and often ingenious considerations it presents, in a very rapid manner, and in many instances wholly unnoticed. Dr. Alard's general conclusions certainly amount to an hypothesis rather than to a theory; but it is an hypothesis that has much of plausibility, and the disquisitions adduced in its support are rendered extremely interesting by the multitude of curious facts,—many of which have been drawn from unmerited obscurity,—which the very extensive erudition of the author has enabled him to bring forward; as well as by the cool, candid, and intelligent manner in which they are conducted.

The proposition argued for in the ninth chapter, is that "*the absorbent vessels of the nervous system draw from the blood the matter of the living solid.*" This chapter involves speculations which, to us, appear to have less of plausibility than any others in the work, and such, indeed, as we do not think congruous with the author's general hypothesis. He thinks that the nerves are the organs first formed in the fetus, and that the nerves "form in their turn all the other organs, by means of the expansion of the numerous filaments which compose them, and which are converted,—we might almost venture to say, which re-appear,—in cellular filaments, muscular fibres; in a word, in the nutritive parencyture of the organs." Why the author deprives the absorbents of powers, in the higher classes of animals, or those possessed of nerves, that he had conceded to them in a general manner, we cannot well discern.* The formation of nerves implies nutrition; which is a sufficient argument against these organs being the essential agents of this function. The priority in respect to the period of their formation to that of the other organs, admitting this antecedency to be proved, is but a feeble argument for the inference that these organs are produced by the nerves. This priority of formation is, however, denied by some emineht physiologists, amongst whom is Carus, who has shown so much accuracy of research in his work on the Nervous System. A pulsating vessel is said, by the physiologist just alluded to, to be the first distinct organ.

The arguments in favour of the proposition which is the subject of this chapter, only, as we think, tend to render it highly probable that the nerves have much influence in the functions of nutrition in the higher classes of animals: whilst it must, at the same time, be considered that nutrition is effected without nerves, not only at the first development of the human embryo, but in fully formed animals; unless we admit, with Oken, that a polypus is a mass in which the nervous matter exists in a state of fusion uniformly extended throughout the animal; but this proposition involves the existence of a faculty without the presence of the organ of that faculty, or it implies the existence of properties in inorganic matter, that are afterwards said to be the peculiar attributes of a certain mode of organization. This sneaking, shuffling way of calling in the Epicurean philosophy to our aid, can never lead to any good: let us profess it openly and candidly, if we do resort to it as the best means of escape from difficulties which perplex us.

---

* "We have seen," says the author, in another place, "that, of the three orders of vessels which compose the bases of the solids of man, the only one to which we may give the denomination of the acting animal, according to the happy expression of Hunter, is that of the absorbents. We have seen that this order of vessels is solely charged with the labour of nutrition; that it presides over growth in infancy, and is alone productive of decrease in old age; that, by means of a property quite exclusive, it draws from the air and from alimentary matters the principles capable of sustaining life; that it is the same vessels which combine them, assimilate them, and distribute them throughout the organs, and repel, by the emunctories, the substances henceforth inappropriate for the exercise of the functions and the maintenance of existence."

In another part of the work, the author speaks of the absorbent system as being "allied, confounded, and identified, with the nervous system." By this last statement, it is true, he may be considered to obviate the charge of incongruity, above advanced; but then it is by, as it appears to us, entering into extremely vague, or at best very hypothetical, and not very plausible, assumptions.

"*The absorbent system is not a whole, the parts of which are connected together, like the sanguineous system; it is composed of several isolated systems;*" is the proposition the author endeavours to establish in the ninth chapter of the work before us. This is so obvious an inference from the views already exposed, that we need not enter into any discussion in order to show in what way it has been arrived at. Having established it, on the grounds already indicated, the author treats, in succession, of the division of the absorbent system charged with transmitting to the blood the lymph and the chyle; the division of the absorbent system charged with the resorption of the fluids which should no longer constitute part of our organs; of the division charged with mingling with the blood the oxygen drawn from atmospheric air; of the division charged with the secretions; of the division exerted in the acts of reproduction and generation. At the same time that the author describes the phenomena dependant on the functions of those several divisions of the absorbents, he finds, in those phenomena, arguments in support of the general hypothesis which it is the principal object of the work to establish. He takes occasion, when treating of the division of the absorbent system charged with the resorption of the fluids which should no longer constitute part of our organs, to enter into a discussion of the opinions which have been advanced respecting the order of vessels by which absorption is effected, in the body generally.

His own notions on this subject must be obvious. As the veins are every-where continuous, in their ramified extremities, with arteries, according to his views, it is impossible that these vessels can be the agents of absorption, unless their parietes are capable of absorbing. The agents of absorption, according to Dr. Alard, are either the vessels ordinarily termed absorbents, or those pellucid vessels which, as he has inferred, spring from the parietes of the blood-vessels, venous as well as arterious, and present their open mouths in every point of the substance of the organs, and on the whole of the membranous surfaces of the body. As we have already remarked, it is only necessary to admit of the existence of such a mode of structure, in order that the difficulties which have beset this subject may be obviated, and the apparently opposing results of different experiments shown to be perfectly congruous and referable to the same mechanism. The facts furnished in the results of injections, noticed in a former part of this article, present, perhaps, the best arguments for the support of his opinions; but physiology and pathology are not wanting in the supply of others from which warranted inferences may be drawn that tend to establish the same propositions. In the first place, Dr. Alard remarks, if we compare all the orifices by which the arterial blood is expended, with the two lymphatic trunks by which it is supposed that all the materials supplied to the blood must pass, we shall see that, if these trunks were the only channels by which the blood could receive supplies, there would soon be no venous blood. This consideration would point out that a considerable proportion of the fluids absorbed pass almost directly into the veins, without entering the thoracic ducts. The greater volumeof the venous than the rterious blood furnishes, also, an argument in favour of the same inference. If, viewing this point in another direction, the veins below the subclavians received only the portion of arterious blood which has not been expended in nutrition, the secretions, and excretions, we should, it seems, find them contain a less volume of fluids than the arteries.

Purulent matter has been found in the abdominal veins, without any signs of inflammation of those vessels, when there has been collections of pus in the belly. This has been frequently observed by Professor Chaussier, especially after peritonitis; but this fact presents, it may be said by the partizans for venous absorption, equivocal indications. The anatomical facts in favour of the inference that pellucid vessels, having orifices opening on the surface of the peritoneum, and communicating with the adjacent veins, and those which seem to show that the venous ramifications are every-where continuous with the arteries, must turn the balance in favour of Dr. Alard's explanation.

Dr. Ribes, who believed in venous absorption when he related* the fact we are about to notice, says that he had conjectured that the lymphatics in the medullary cavity of the bones, which absorb the marrow, terminated in veins; and experience has partly verified his conjecture, by showing that, by injecting the hepatic veins, he could fill the superficial lymphatics of the liver. We have before noticed this point of anatomy; and shall therefore only add now, that this experiment, repeated several times with the same success, satisfied him that all the vessels of the order of lymphatics do not terminate in the thoracic duct. After the discovery of such a fact, it seems strange that the phenomena which at that time were considered to prove venous absorption, should any longer be thus interpreted. Dr. Magendie thinks he has obviated these equivocal indications by some recent experiments, and proved that venous trunks absorb by their parietes. We shall elsewhere notice his memoir on this subject.

The only circumstances that have been brought forward as proofs of the ramifications of veins presenting open mouths on the surfaces of the membranes and the cavities of the cellular tissue, are that fluids injected by the veins have been effused in those parts. But Dr. Ribes, who relates these facts, acknowledges that such results were to be obtained only when the experiment was made on a body in which putrefaction had commenced; and it seems, hence, so highly probable that rupture of the minute venous ramifications took place, so as to render this argument one of very little force in favour of venous absorption, or the communication of the cavities of those vessels with extraneous surfaces in the natural state of the parts.

The views of Dr. Alard seem to be alone qualified to explain the promptitude with which a considerable quantity of certain drinks sometimes passes from the intestinal canal to the urinary passages; how it happens that asparagus, prussiate of potash, and many other substances, when introduced into the stomach, soon manifest signs of their presence in the urine, whilst the venous blood, at least that

* In the eighth volume of the *Memoires de la Societé Medicale d'Emulation.*

drawn from the arm, does not appear to contain them, or to have suffered any alteration;—how a liquid charged with odorific principles, introduced into the abdominal cavity, immediately manifests itself in the veins; whilst an analogous liquid passed into the intestines, promptly appears in the lacteals, and remains a stranger to the veins;—how the milk possesses the odour and other predominant qualities of food and other substances received into the stomach, (by which a child is purged when its nurse has taken a cathartic;) whilst the blood and other secretions have no such odour or qualities;—and, lastly, how certain substances, as milk, water pure or mingled with turpentine, inserted in a wound of the integuments, penetrate the trunks of the valvular absorbents, and not into the veins; whilst certain other substances, as the *upas tieuté*, for example, inserted in the same manner, pass rapidly into the veins, and do not appear in the valvular absorbents.

All these phenomena are readily explicable, if we admit the existence of absorbent lymphatics every where arising from the textures, and terminating in veins; so that little centres of absorption are formed, each of which, being modified according to the arrangement of the parts which give origin to it and the diversity of the exciting agents affecting it, directs the absorbed matters to the veins, the lacteals, or certain excretory organs; whilst diversities in the sensibility, at different times and in different parts, enables them to exert that specific action by which they select and transmit certain matters, and refuse to receive others of a different kind.

Details on the rest of the points treated on in the divisions above enumerated, do not come within our intentions: we pass on, therefore, to the next chapter, in which the author treats of the structure and physiology of the mucous membranes, the cellular tissue, and the skin. The author's peculiar notions of the structure of these organs must be obvious, from what has been stated in former parts of this article. Excepting his assimilation of the functions of their capillary vessels to those of the absorbents, commonly so termed, his physiological considerations are characterized by a judicious exposition of what had already been advanced, rather than by any remarkable novelty. He dwells much upon the similarity of the structure of the skin and mucous membranes, and the analogy, in many respects, between their functions. We find some of the most remarkable proofs of the latter assertion in the lower classes of animals: the zoophyte which has the form of the finger of a glove, (the *hydra fusca*,) for example,—the interior of which is its digestive organ,—may be turned inside out, and yet the animal will live, having the functions of its two surfaces reversed.

This, and the two ensuing chapters, in which the author treats of the development of animal heat,—on the influence of the absorbent vessels on the establishment of the constitutions proper to each period of life,—and on the modification of temperaments in individuals,—seem to have been introduced in order that the account of the physiology of the absorbent system given in this work might be rendered complete, rather than with the intention of proposing any thing novel of remarkable importance. His disquisition on the temperaments is, as he acknowledges, principally derived from the writings of Lorry, Cabanis, and Halle; with which we suppose our readers are well acquainted.

The subject just mentioned terminates the first volume of the work; the second will become the subject of an article in the ensuing Number of this Journal.

---

*Principes géneraux de Physiologie-Pathologique, coordonnés d'aprés la Doctrine de M. Broussais.* Par L. J. Bégin. 8vo. pp. 390. Mequignon-Marvis, à Paris, 1821.

[In continuation from page 345.]

The fifth chapter of the work of Mr. Bégin treats of *the local effects of irritation.* On this subject Mr. Bégin advances nothing of considerable importance that has not been stated in this Journal, in the articles professedly devoted to the doctrine of Broussais.

Mr. Bégin labours hard to support the doctrine of Broussais respecting the origin of morbid growths and alterations of structure;† and he seems to be well aware that he has undertaken a perplexing task. With respect to the origin of all morbid tumors from irritation, (in the sense in which Mr. Bégin uses this term,) there is the strong objection, that such tumors are not unfrequently found in the lungs and other organs of new-born children: and then we are perplexed to the utmost, when we attempt to imagine how an augmentation of vital action simply, differing only in being more or less in degree, can produce the numerous varieties of morbid structure witnessed in the human body.

We pass over this vague and unsatisfactory disquisition to the next chapter, which treats of *the general effects of irritation.* In this chapter, also, there is but little to notice that has not been already considered in this Journal: the value of the author's discussions consists chiefly in details which cannot be comprised in the limits of this Journal. Mr. Bégin, however, we should not neglect to remark, has combatted very forcibly for the truth of the most essential points in the hypothesis of Broussais relative to fever, though he differs from him in some points; and especially in regard to the order in which the phenomena of this condition of the economy are developed. Broussais seems disposed to infer that fever, when it does not arise from primary irritation of the stomach and small intestines, takes place from irritation affecting those organs sympathetically from disease of some remote part. He thinks that diseases in remote parts,—as, for example, a severe wound in one of the legs, or a phlegmon in the arm,—does not excite fever, unless they sympathetically produce such irritation; and he is disposed to believe that the heart and the brain are

---

* See the Numbers of this Journal for January and February 1820, for details on this subject.

affected, in the development of fever, subsequently to the stomach. Mr. Bégin thinks that sometimes one of those organs is affected first in order, and sometimes another: he considers, however, that a knowledge of the order of those affections is not a matter of so much importance as Broussais and some of his partizans have imagined; and that what is of greatest consequence to the physician, and that which he should always bear in mind, is that, "whenever there is fever, there exists irritation, more or less considerable in degree, of the mucous membrane of the stomach, the heart, and the brain.

Mr. Bégin dwells much, with good reason, on the influence of civilization, and the great excitement of the nervous functions which accompanies its progress, in the origin and development of diseases. "We often find, for example," he says, "according to the accounts of travellers, that the most extensive and painful wounds hardly attract the attention of the savages of North America or New Holland; and, if abundant suppurations, or other consecutive accidents which depend on the nature of the affected parts, do not cause them to perish, they become cured spontaneously and with facility, without having been sick. Their internal diseases present the same simplicity, and an analogous absence of any considerable sympathetic accidents." Amongst other illustrations of this point, derived from sources more near to us, he relates the following anecdote.

"On leaving Moscow, in the disastrous campaign of 1812, the tenor of my sensations led me to make an excursion over the fields which were one of the latest and most brilliant theatres of our military glory,—the fields on which had been fought the battle of the Moscowa. The villages which had surrounded them were all destroyed; the calmness of death reigned over those plains where, only a few days previously, six hundred thousand men disputed a victory, the consequences of which were to become so fatal to the welfare of France. Our army, already overwhelmed with ills of every kind, passed in silence, and almost without recognizing them, the very places which had been rendered immortal by its valour. At the extremity of the open country bordering the road, and near to the confines of a forest, I was suddenly roused from my reverie by cries of lamentation that seemed to come from a spot very near to me. I looked around in vain; I could see nothing but putrefying bodies. The complaints, however, continued, and I could not then doubt that a living man was hidden amongst the adjacent ruins. Having descended from my horse, I, after much pains and long researches, discovered, at the bottom of the ditch bordering on a bastion, and in the interior of a horse, a Russian soldier, whose leg had been carried away by the charge of a cannon. The unfortunate man had escaped the attention of those who, soon after the battle, had gone over the field for the succour of the wounded; and he had remained there for six weeks, finding in the body of the animal his food and habitation. He had deprived the ribs of their flesh, and removed the internal parts, and thus converted the thorax into a sort of cage, still enveloped with the skin, in which he lay in a half-recumbent posture. An abundance of pus flowed from his wound, and added to the stench of the carcass on which he fed,

Several other horses, lying near to that in which he was situate, bore marks of his voracity; large pieces of them having been detached by means of an old knife which he had about him. This man seemed to be hardly sensible of the danger he had encountered. He was pale and meagre, but his strength appeared to be but little diminished; and his movements were characterized by firmness and assurance. The surface of the stump, which was exposed, was unequal, but covered with granulations of a favourable appearance."

On comparing this case, Mr. Bégin says, and others of a similar kind, with those presented by the practice of great cities, we are tempted to believe that they have occurred in subjects of quite a different species.

Mr. Bégin discusses in this chapter, besides the phenomena of the several forms of fever, termed by nosologists idiopathic, inflammatory, synochal, typhous, eruptive, &c.—all of which, he argues, are connected, as effects, with irritation of the mucous membranes of either the alimentary canal or the respiratory organs,—dyspepsia, (which he attributes, generally, to chronic inflammation of the mucous membrane of the stomach, (a point of doctrine that Dr. Parry has done much for the establishment of in England,) dysentery, (from irritation of the mucous membrane of the large intestines,) tabes mesenterica, scrofula, phthisis; in conformity with views of Broussais that have been already exposed in this Journal, in which they are all traced to irritations of some one or other of the mucous surfaces.

Diseases of the heart are next considered, without, however, any thing remarkably novel being adduced. After this, Mr. Bégin treats on the more chronic forms of inflammation. Although less violent than those produced by acute inflammations, the disorders caused by chronic irritations are not less remarkable, and do not present a less degree of interest. Persons affected with chronic gastro-enteritis, are exposed, Mr. Bégin says, to the greater part of the forms of mental alienation; mania itself is very often referrible to this cause; melancholy and hypochondriasis almost always depend on it. Mr. Bégin thinks that chronic inflammation produces mental affection as it is seated in different organs. Individuals having chronic gastro-enteritis readily despair of their life; many of them feel an horror of existence, and put an end to it by suicide; "whilst others, a prey to constantly-renewed fears, dread the action of all external bodies, and regard with terror the approach of death, which they consider as inevitable." Chronic lesions of the lungs produce opposite effects on the intellectual functions: persons in phthisis preserve, to the last instant of their existence, the full powers of their intellects, and expire in the midst of preparations for future enjoyment. Organic affections of the heart exert a peculiar influence on the mental faculties: the subjects of them seem to fear all acute moral impressions, which cause disturbance of the circulation, and aggravate the evils which accompany them. Men who once evinced the most undaunted courage, become, on suffering disease of this organ, timid in the extreme, and cannot support even the idea of the smallest danger.

A point of very great importance in the history of irritations in general, and especially of chronic irritations, is the disposition they have, in certain cases, to occur in a periodic manner after regular intermissions.

"In what do intermittent differ from continued irritations?" is a question which Mr. Bégin discusses, as one of very great importance in regard to the etiology of intermittent fevers. If we consider, he says, the organ which is the seat of the former, during the paroxysms of their accession, or if we examine the general phenomena they produce, it is not possible to discover any difference which may serve to distinguish them from the latter. Ophthalmia, cephalalgia, erysipelas, rheumatism, &c. do not differ in any respect, when they are of an intermittent kind, from what they are when they are continued; and there exists between the periodicity of their occurrence and their absolute continuity, a multitude of shades which connect the two conditions. The excitement is sometimes too intense to be dissipated otherwise than very imperfectly: we then observe simple alternations of exacerbation and remission. At other times the diminution of the morbid action is more considerable, and the return of the paroxysms is marked by a rigor of greater or less severity; the disease is then termed remittent. In some other varieties, the "vital concentration" has hardly diminished, when it is reproduced, and develops another paroxysm; and, lastly, the intermission may be complete, and vary in duration from the period of a few minutes to that of two, three, or even seven or more days; and hence the multitude of distinct species of fevers of this kind arranged in the tables of nosologists. All these conditions are closely allied, and we may frequently, by stimulating the affected organs, transform a quartan fever, for example, into a tertian or quotidian, or even approximate the paroxysms so that it will be regarded as a continued fever; and, in all these cases, the morbid phenomena undergo no other sensible change than that of not disappearing at certain epochs.

The periodical returns of intermittent irritations in the same textures, when prolonged for a considerable period, produce disorganizations similar to those which are the results of continued chronic phlegmasiæ. Indeed, although perfectly intermittent during its early stages, the inflammation, on being frequently renewed, gradually becomes continuous: each accession adding to the stimulation, the organ returns in a less complete manner to its natural state. It remains red and irritated during the apyrexia; but this excitement is but little remarkable at first, because the slight degree of phlogosis it determines is hardly manifest after the state of intensity which accompanies the paroxysms. It is only after a long time, and when the local disorder is very extensive, that the symptoms no longer, in appearance, completely disappear, and that we witness the signs of the disorganization of the tissues. It is easy to trace precisely all these gradations, when the disease affects the exterior parts of the body; and we may acquire, by means of the general symptoms, almost as satisfactory knowledge of their succession when they have their seat in the viscera. In cases of intermittent fever, we find, after their prolonged duration, the mucous membrane of the stomach thickened, dark-coloured, and more or less disorganized. The liver and the spleen,—which almost always participate with irritations of the stomach and duodenum, and which are, in each paroxysm, the principal seats of sanguineous concentration,—become affected with chronic engorgements or secondary chronic inflammations. The mesenteric glands tumefy, and suffer change of their structure, in the same manner as if the intestinal irritation were continuous.

Besides the local effects above considered, intermittent fevers occasion very remarkable phenomena in the economy more generally. The first period of each paroxysm is accompanied by the concentration of the vital actions on the digestive organs; the blood, directed to the interior parts, engorges the liver, the spleen, the lungs, and reddens the irritated mucous surfaces; the heart is surcharged, and exerts itself to propel the fluid which accumulates in the large vessels, from the small vessels of the exterior parts being devoid of blood: hence results derangement of the respiration and circulation, and, as a consequence, disorder of all the other functions. The lungs especially gradually contract chronic irritation of their mucous membranes, which not unfrequently leads to phthisis. The heart suffers, in some cases, more or less extensive dilatation of its parietes, especially of those of the right auricle and ventricle. The circulation and respiration being thus periodically disturbed for several hours, the process of sanguification becomes less perfectly effected; accumulations of serum take place in the cavities of different parts of the body; digestion is effected with difficulty; the reparatory fluids become less abundant than is necessary, or possess ill qualities; and hence emaciation, debility, and sometimes all the phenomena of scurvy.

The alterations which the fluids undergo, from derangements of the organic functions, constitute the subjects of some discussions by Mr. Bégin, and he expresses his intention of considering this part of pathology more fully at a future period, in another work. At present he produces but little more than an obvious development of the notions of Broussais already stated in this Journal, with some additions, however, relating to the existence of more or less acrid secretions in cases of irritations of the organs, and to the generation of specific poisons; which comprise, of course, nothing more than a history of known effects; the causes of those changes being inscrutable by the senses.

[We have yet to notice the author's therapeutical disquisitions, which will form the subject of an article in the next Number.]

